# Anterior lamellar corneoscleral keratoplasty for the management of corneal thinning following treatment with mitomycin-C and interferon to treat highly suspected ocular surface squamous neoplasia: a case report

**DOI:** 10.5935/0004-2749.2023-0027

**Published:** 2024-07-09

**Authors:** Paulo Phillipe do Vale Ricardo Moreira, Daniele Longo, Marcella Polisuk, Tamires Able Carmona, Renato Ambrosio Jr

**Affiliations:** 1 Centro de Estudos e Pesquisas Oculistas Associados, Rio de Janeiro, RJ, Brazil; 2 Hospital Federal de Bonsucesso, Rio de janeiro, RJ, Brazil; 3 Universidade Federal do Rio de Janeiro, Rio de Janeiro, RJ, Brazil

**Keywords:** Corneal diseases/diagnosis, Carcinoma, squamous cell, Conjunctival neoplasms/surgery, Interferon-alpha/therapeutic use, Mitomycin/therapeutic use, Ophthalmic solutions/administration & dosage, Humans, Case reports

## Abstract

A patient presented with corneoscleral thinning five months after the treatment of suspected ocular squamous surface neoplasia with mitomycin-C and interferon. For tectonic and aesthetic purposes, we decided to perform lamellar corneoscleral transplantation. The approach used established new tectonic support and corneal homeostasis. This technique might be an option in similar cases.

## INTRODUCTION

Ocular surface squamous neoplasia (OSSN) describes neoplastic abnormalities of the conjunctiva and cornea, including dysplasia, carcinoma in situ, and squamous cell carcinoma (SCC)^(1)^. Mitomycin-C (MMC) is a widely used OSSN treatment but reported side effects include conjunctival hyperemia, allergic reaction, punctate keratopathy, punctal stenosis, and photophobia, most of which are relatively minor and reversible^(2)^. However, a critical potential risk is corneoscleral thinning and perforation. Therefore, in this study, we demonstrate a technique that facilitates the reversal of the thinning process through the insertion of new tissue with collagen matrix-producing cells.

Although surgical excision using the “no-touch technique” is the standard treatment for OSSN, nonsurgical topical treatment and intralesional chemotherapy have both become increasingly significant. Chemotherapeutic agents such as interferon-alpha-2b, (lFN-α2b), 5-fluorouracil (5-FU), and MMC, have been found effective as either primary or adjuvant therapy for OSSN. Compared to excision and cryotherapy, primary topical chemotherapy is relatively noninvasive, treats the entire ocular surface, and avoids the risks of surgery^(3)^.

In this study, we illustrate a potential management strategy for corneal thinning in OSSN patients.

## CASE REPORT

**Figure 1:**
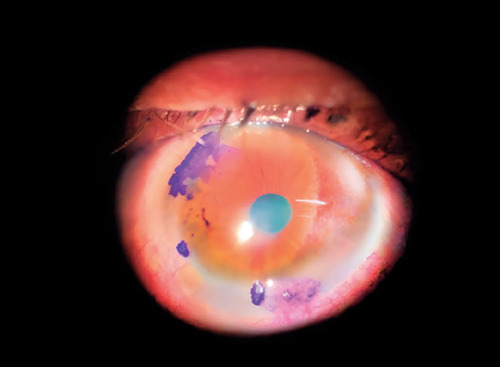
Staining with 1% toluidine blue revealed three lesions of the perilimbal conjunctiva in our patient. These were in the superior, temporal, and inferior regions (6h, 8h, and 11h).

A 69-year-old man diagnosed with T-cell cutaneous lymphoma and ocular rosacea was referred to the Department of Ophthalmology of our tertiary center due to lesions in the ocular periphery of the right eye (OR). The patient had a best-corrected visual acuity of counting fingers at 3 m in the OR and 1 m in the left eye (OS). Using 1% toluidine blue staining, we found three lesions of the perilimbal conjunctiva in the superior, temporal, and inferior regions (6h, 8h, and 11h) (Figure 1). We began neoadjuvant therapy with lFN-α2b (1.000.000 Ul) every 6 h for four months to avoid more than 180° limbal dysfunction.

After one month, topical 0.1% fluorometholone acetate and 0.02% MMC every 6 h for 14 days were prescribed due to optical coherence tomography (OCT) findings suggestive of cancer cell invasion beyond the epithelium. The MMC was suspended for 7 days and then another 7-day cycle was performed.

The lesions regressed over two months, leaving a small stained area. Therefore, the scheduled excision was not performed. At follow-up, conjunctival hyperemia and total epithelial defect were observed. We suspected an MMC lesion and discontinued its use. We performed an impression cytology biopsy, which showed a strong inflammatory reaction on the ocular surface due to the recent use of topical MMC, with residual effects on the epithelium. The sample was negative for dysplastic or neoplastic cells.

Due to persistent epithelium defect and scleral show, the patient was referred to the Oculoplastic Department. However, no surgery was required.

Five months later, the patient returned with moderate corneal edema, conjunctival hyperemia, hypopyon (2.7 mm), ciliary injection, and a corneal ulcer covering nearly all of the cornea (5.7 mm × 4.7 mm), with corneal melting and inferior corneoscleral thinning. Treatment with gentamicin (4%), vancomycin (5%), and atropine (1%) produced no improvement. Medroxyprogesterone was also prescribed; however, the patient did not adhere to this.

**Figure 2:**
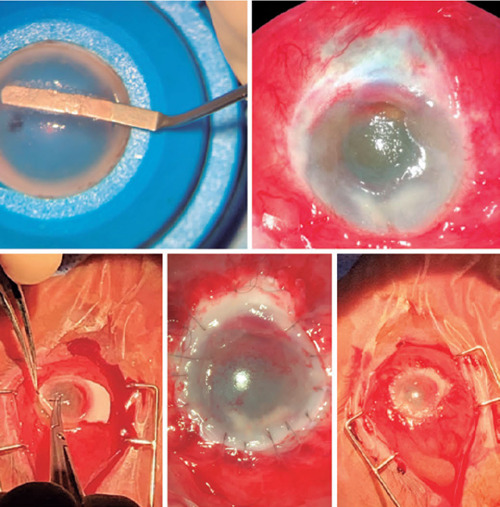
The donor cornea preparation procedure, in which the endothelium faces upwards and the epithelium, downwards. An incision was performed using a micrometer knife at 100 µm, and the deep stroma was separated using a DSEK spatula.

Although we observed improvement in the infection, a tectonic corneoscleral lamellar anterior transplant was required due to extreme thinning of the cornea and inferior sclera. A partial transplant was performed using a donor cornea, which was placed upside down in the artificial chamber with the endothelium facing upwards (Figure 2). This was achieved using a micrometer diamond knife entering at a depth of 100 µm as peripherally as possible. The lamella was separated at this depth and cut toward the periphery. The sclera was then detached from the cornea through the limbus using scissors, leaving a sclera pedicle with a base of 4 mm and a width of 2 mm. The receiving cornea was prepared by manually removing the epithelium with a type-15 scalpel. The donor cornea was positioned over the recipient’s cornea and sutured through the limbus with superficial corneal sutures to the donor cornea and deep in the recipient’s limbus to allow perfect positioning and epithelial migration. The donor sclera was similarly sutured over the thinned recipient sclera and covered with the patient’s inferior conjunctival epithelium.

In the first month after surgery, the patient had no symptomatic complaints, with conjunctival hyperemia in the lower third and thinning of the subjacent inferior sclera. The transplant adhered well, with adequate tension of the corneal sutures and mild edema. The patient’s BVCA was <0.1 and bidigital tonometry (BDT) findings were normal. Central corneal pachymetry as measured by OCT was 670 µm. B-ultrasound showed thickening of the posterior pole and posterior vitreous detachment, with no hyperreflective focus.

**Figure 3:**
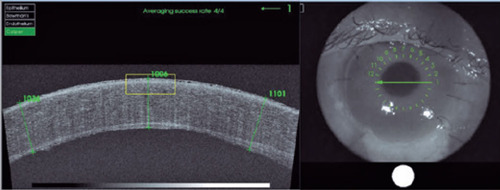
Optical coherence tomography images showing corneal thickness and the remaining interface. As shown, there was no epithelial growth in our patient. Corneal edema was evident due to endothelial injury.

At the four-month follow-up, the patient continued to have no complaints. However, biomicroscopy and OCT (Figure 3) showed worsening of the corneal edema, epithelial microcysts, and reactive miosis. Scleral thinning was maintained. Eye closure was preserved, with no scleral show. The BDT and BVCA measurements were maintained. A triple procedure for ocular rehabilitation was indicated but the patient declined.

## DISCUSSION

Topical lFNα2b has been increasingly favored for OSSN treatment over the past 15 years due to its low toxicity, effective OSSN regression outcomes, and recurrence rates similar to those of surgery^(4)^. When performing extensive biopsies of OSSN cases, it is important to be cautious of potential complications, including limbal stem cell failure.

IFNα2b is an endogenous glycoprotein released by several immune cells. lt has antiviral, antibacterial, immunomodulatory, and antitumor properties. MMC is an antibiotic isolated from Streptomyces caespitosus broth that exerts significant antitumor effects. MMC acts as an alkylating agent in all cell cycle phases, producing efficient local chemotherapeutic action^(4)^. Despite the effectiveness of these two drugs, their use does not come without risks. Blasi et al. have shown that many patients treated with MMC complain of ocular discomfort, conjunctival hyperemia, chemosis, and corneal epitheliopathy. A comparative study has identified complications associated with subconjunctival lFN, including transient fever and myalgia^(5)^.

The patient presented in this study had cutaneous T-cell lymphoma and rosacea, possibly leading to a state of immunodeficiency. Combined with chemotherapy, this may have contributed to the development of keratitis and corneal thinning. Despite the resolution of the infection, progressive thinning occurred, indicating a lack of matrix production with worsening corneal homeostasis. This led to the need for a transplant.

The transplantation surgery performed in this case aimed to add collagen matrix-producing cells with new keratocytes from a donor. The transplant led to an optical refractive result with a reorganization of the corneal and scleral structure, preventing perforation and improving surface quality.

We aimed in this paper to describe a treatment option for corneal thinning in the absence of perforation, in which a tectonic transplant can improve corneal homeostasis and avoid perforation while maintaining some optical functionality. We also aimed to increase awareness of the risks involved in the use of MMC and lNF as they can lead to serious side effects. Further studies are required to improve our understanding of this.
